# Supplementary material for: The Root-Colonizing Endophyte Piriformospora indica Supports Nitrogen-Starved Arabidopsis thaliana Seedlings with Nitrogen Metabolites
Source: Int J Mol Sci. 2023 Oct 19;24(20):15372. doi: 10.3390/ijms242015372 (PMC10607921; doi:10.3390/ijms242015372)
Supplement: Supplementary file 1 [file ijms-24-15372-s001.zip › ijms-2630019-supplementary.pdf]

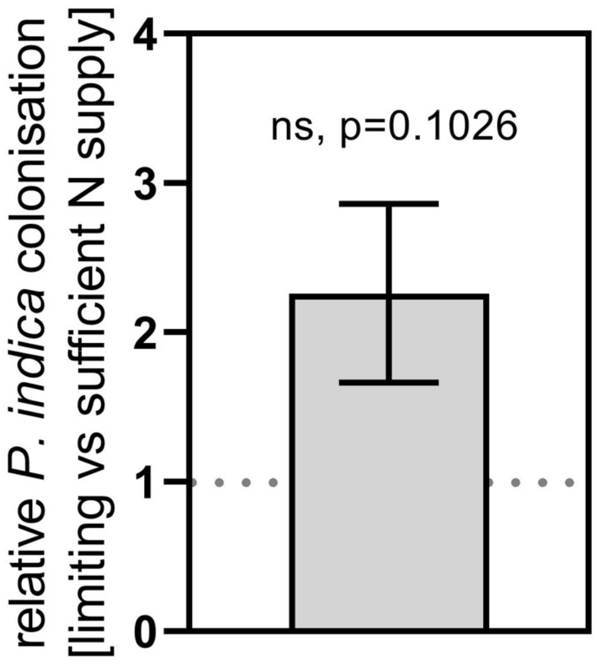

Figure S1. ***P. indica*** **colonization of** ***A. thaliana*** **WT roots under different N conditions.** Shown is the ratio of fungus colonization of plants grown on N-free medium (-N) *vs.* full medium (+N) after 5 days of co-culture, N=3. Fungus colonization was analysed by comparing expression of the fungus housekeeping gene vs. the plants housekeeping gene, see methods for details. Statistics analyses was done by t-test and the obtained p value is indicated in the graph. The raw data (Δ CT) can be found below.

	Δ CT PNM+N (*PiTEF1 - AtRPS18B*)	Δ CT PNM-N (*PiTEF1 - AtRPS18B*)
Replicate 1	8.44	6.60
Replicate 2	6.30	5.92
Replicate 3	6.24	5.24
**Average**	**6.99**	**5.92**

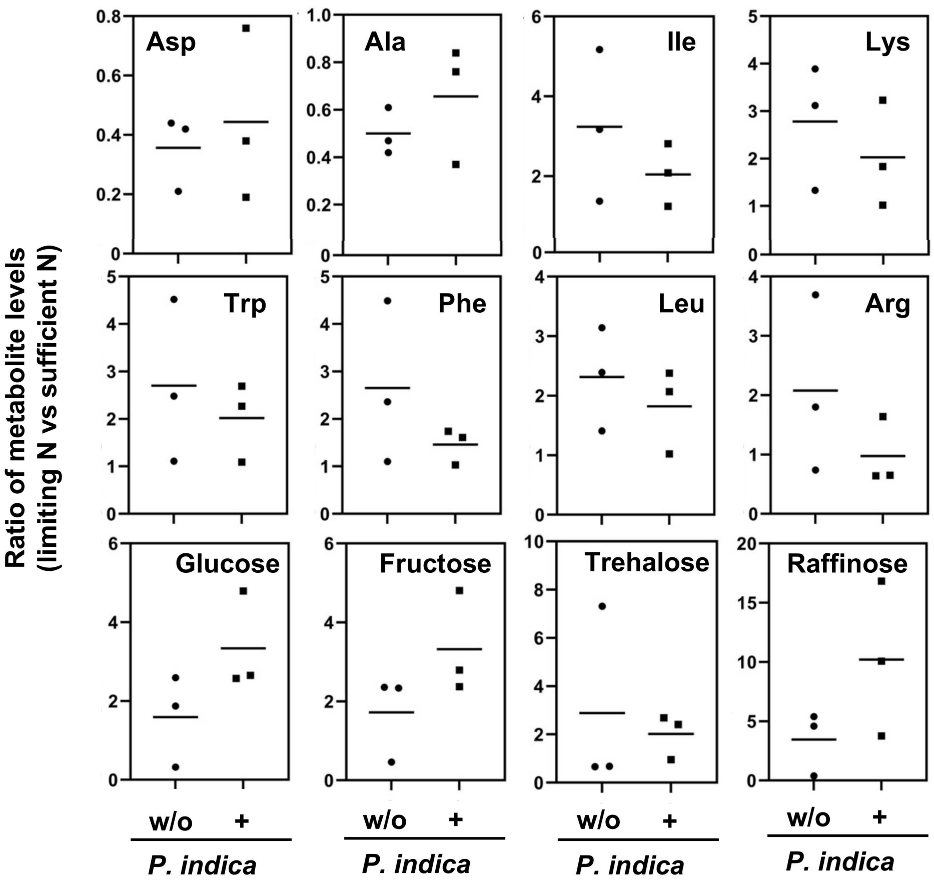

Figure S2. Selected differentially accumulated metabolites (DAMs) in Arabidopsis shoots. Values are given as the ratio of the (relative) metabolite content in N-limiting to N-sufficient growth condition as measured by GC-MS. Data from 3 independent cultures on pools of 20 plantlets. For each of the two conditions (without (w/o) and with (+) *P. indica*, the 3 data points correspond to the ratio of independent replicates each consisting of 20 plantlets.The mean value of the three replicates is indicated by a tray. Statistical analysis was performed, none of the comparison is significant to a p-value of 0.1.

Supplemental Table S1. Content of 129 metabolites in Arabidopsis Col-0 plantlets grown under limiting and non-limiting nitrate supply and in absence (w/o) or presence (+) *P. indica* for 2 days.

Data were obtained by GC-MS . Data of 3 independent experiments (#1,#2,#3) are given, values underlined in gray are below detection level and not taken into account.

NB: data correspond to 20 plants for each point

compound	unit	non-limiting N_ w/o P.i._#1	non-limiting N_ w/o P.i._#2	non-limiting N_ w/o P.i._#3	non-limiting N_ + P.i._#1	non-limiting N_ + P.i._#2	non-limiting N_ + P.i._#3	limiting N_ w/o P.i._#1	limiting N_ w/o P.i._#2	limiting N_ w/o P.i._#3	limiting N_ + P.i._#1	limiting N_ + P.i._#2	limiting N_ + P.i._#3
2-4-dihydroxybutanoate	(arbitrary/mgFW)	6,60E-10	6,47234E-05	0,000100076	6,60E-10	5,24971E-05	3,20119E-05	7,80671E-05	7,58209E-05	7,66068E-05	8,4969E-05	0,000126737	7,5178E-05
2-Oxoglutarate	(µg/mgFW)	0,02263165	0,037744485	0,019960429	0,023863575	0,038468356	0,02030512	0,009990877	0,019789102	0,032400424	0,008247285	0,02324972	0,016279076
4-Hydroxycinnamate-trans	(arbitrary/mgFW)	5,24774E-05	1,00161E-05	4,66727E-05	4,44021E-05	5,16382E-05	6,75315E-05	2,02826E-05	6,60E-10	7,75097E-05	3,14395E-05	0,000193649	7,81466E-05
4-hydroxyproline	(arbitrary/mgFW)	0,000463143	0,000370289	0,000532327	0,000524177	0,000385549	0,000713582	0,000431421	0,00057605	6,60E-10	0,000459965	0,000633944	0,000638811
5-(methylthio)pentanenitrile	(arbitrary/mgFW)	0,000907059	0,001830281	0,001268647	0,00092124	0,001494324	0,001767171	0,000649566	0,001065172	0,002147919	0,000468818	0,001393238	0,001388276
5-Ethyl-Glucopyranoside	(arbitrary/mgFW)	0,098392316	0,005753347	0,013617531	0,099135464	0,002517892	0,003277737	0,106959012	6,60E-10	0,019565286	0,115563893	0,001682499	0,001821398
a-Aminoadipate	(µg/mgFW)	0,003096904	0,001475597	0,002758092	0,003696834	0,000941962	0,003611818	0,00323313	0,001101597	0,006007307	0,003159699	0,001474643	0,003651572
Aconitate	(µg/mgFW)	0,001400758	0,002614509	0,001405194	0,001715173	0,002222568	0,000953721	0,000161576	0,000586067	0,000400118	0,001108845	0,000356641	0,000380399
Adenosine-5-P	(arbitrary/mgFW)	5,20568E-05	7,04009E-05	6,74734E-05	7,1811E-05	4,74434E-05	7,47205E-05	8,05615E-05	0,000129111	0,000102103	7,48951E-05	8,13915E-05	0,000106216
Agmatine(-NH3)	(µg/mgFW)	0,016576997	0,028332897	0,056506701	0,012079406	0,034065263	0,078585224	0,030732036	0,031112901	0,110413946	0,029347219	0,027586711	0,074590902
Alanine	(µg/mgFW)	0,031068526	0,041322105	0,033054912	0,040091205	0,033213514	0,030015899	0,012903383	0,025413556	0,015480775	0,014917131	0,02792454	0,022866059
Allantoin	(arbitrary/mgFW)	0,000615209	0,000217231	0,001276232	0,000270399	0,000493864	0,001367112	0,001153229	6,60E-10	0,003461138	0,000169572	6,60E-10	0,001617712
alpha-Tocopherol	(µg/mgFW)	0,00574905	0,005660253	0,005514558	0,008033111	0,003722036	0,005862751	0,007542847	0,00966572	0,006876469	0,01036791	0,009673792	0,009752888
Anhydroglucose	(arbitrary/mgFW)	0,000902586	0,000947596	0,000824403	0,001392114	0,001216137	0,000988311	0,001309979	0,000796124	0,000867315	0,001329044	0,001182931	0,000988937
Arabinose	(µg/mgFW)	0,00117073	0,001303759	0,001145916	0,001289138	0,000955695	0,00120167	0,001450875	0,002045981	0,001041893	0,001630109	0,002105433	0,001480152
Arginine	(µg/mgFW)	0,02027045	0,016809383	0,064832426	0,008988709	0,019363608	0,078133569	0,036396153	0,012440527	0,239444846	0,014742234	0,012638762	0,050323812
Ascorbate	(µg/mgFW)	0,003034944	0,003388038	0,004271462	0,00366602	0,002784647	0,003158757	0,002979814	0,004480351	0,003404	0,003515383	0,00447271	0,004193255
Asparagine	(µg/mgFW)	0,030099734	0,031984828	0,057242932	0,029939919	0,029149151	0,060030367	0,029124193	0,022761758	0,113839149	0,015198184	0,018674202	0,052316816
Aspartate	(µg/mgFW)	0,081330713	0,093210573	0,078516449	0,092732595	0,099163846	0,063315329	0,017438272	0,039498312	0,034360917	0,017517334	0,037624584	0,047977959
b-aminoisobutyrate	(µg/mgFW)	0,000109947	8,6383E-05	0,000227306	0,000192261	6,60E-10	0,000230536	6,60E-10	6,60E-10	0,000515994	6,60E-10	6,60E-10	0,000194639
beta-Alanine	(µg/mgFW)	0,002085439	0,00179569	0,002287567	0,002057	0,0013178	0,002139401	0,002675143	0,002025816	0,00592107	0,002450955	0,00194658	0,002339435
beta-indole-3-acetonitrile	(arbitrary/mgFW)	0,007997851	0,007220153	0,00588044	0,008755324	0,005328633	0,005513505	0,009680886	0,008930813	0,005173053	0,008740346	0,007320801	0,006389157
beta-Sitosterol	(µg/mgFW)	0,143871466	0,159134878	0,143235425	0,15596023	0,136246348	0,173810233	0,161462733	0,213624594	0,157370272	0,174751326	0,198952189	0,177007392
beta-Tocopherol	(arbitrary/mgFW)	3,72668E-05	2,32419E-05	6,60E-10	5,13954E-05	1,96729E-05	2,70384E-05	4,37811E-05	4,51488E-05	2,28887E-05	6,18686E-05	5,19347E-05	4,02566E-05
bis-glycerol-P	(arbitrary/mgFW)	6,60E-10	0,001799184	0,001994561	6,60E-10	0,001452918	6,60E-10	6,60E-10	0,002142973	0,001427164	6,60E-10	0,002692487	0,001997802
Campesterol	(arbitrary/mgFW)	0,001043419	0,001117079	0,000874065	0,001165467	0,001005456	0,001182078	0,001205609	0,001516964	0,001149196	0,001234051	0,001448014	0,001534352
Cholesterol	(µg/mgFW)	0,000263128	0,00045878	0,000630258	0,000596783	0,000368127	0,000506489	0,000360877	0,001050652	0,000372099	0,000492423	0,000636591	0,00058605
Citramalate	(µg/mgFW)	0,0016544	0,002969394	0,002132239	0,001939635	0,002623295	0,001487573	0,000300894	0,001265963	0,000776675	0,000301004	0,001199617	0,001124047
Citrate	(µg/mgFW)	0,086269234	0,182038343	0,092083277	0,121788484	0,155028258	0,047337091	0,007308611	0,040258563	0,017959381	0,008804813	0,04592844	0,019509247
Cys	(arbitrary/mgFW)	0,000250467	0,000260179	0,000248934	0,000240454	0,000235026	0,00030046	0,000319407	0,000269124	0,000507778	0,000268593	0,000302	0,00036764
Dehydroascorbate	(arbitrary/mgFW)	0,013931895	0,007187382	0,008693245	0,014842585	0,005055373	0,007397534	0,013876558	0,009670579	0,007748314	0,015827961	0,008847437	0,008276699
Digalactosylglycerol	(arbitrary/mgFW)	0,003739732	0,006740487	0,004126346	0,004896684	0,004111925	0,00259377	0,008897466	0,018333721	0,002014169	0,015142957	0,020059839	0,006130051
Epicatechin	(µg/mgFW)	6,60E-10	6,60E-10	6,60E-10	6,60E-10	1,30793E-05	6,60E-10	6,60E-10	6,60E-10	7,93716E-05	6,60E-10	8,64411E-05	6,60E-10
Erythritol	(µg/mgFW)	0,00106654	0,000713929	0,000719366	0,001129899	0,000341541	0,00032718	0,00104775	0,000767071	0,000907136	0,000772298	0,00053716	0,000346248
Erythronate	(arbitrary/mgFW)	0,000882972	0,001241167	0,001116348	0,001006922	0,000990004	0,00090216	0,000322193	0,00096546	0,000658028	0,000329648	0,000980519	0,000910436
Ethanolamine	(µg/mgFW)	0,006072868	0,010717958	0,007763208	0,005625867	0,004903393	0,007247889	0,007035288	0,008974523	0,006180056	0,006593405	0,01095801	0,007100017
Ethanolamine-P	(arbitrary/mgFW)	0,00029078	0,00015385	0,000343231	0,000274132	0,000332008	0,000481644	0,000189066	0,000173979	0,000562582	0,000276043	0,000315302	0,000459332
Ethylphosphate	(arbitrary/mgFW)	1,6807E-05	3,85902E-05	1,12058E-05	6,03361E-06	6,60E-10	3,67236E-05	1,32211E-05	6,16548E-05	8,2165E-06	1,18507E-05	0,000120192	2,80575E-05
Ferulate-trans	(µg/mgFW)	0,002026395	0,001451464	0,001730747	0,001384046	0,001458009	0,00090453	0,000613516	0,000659645	0,000843558	0,000599523	0,001320262	0,001009384
Fructose	(µg/mgFW)	0,270128155	0,422014679	0,24300799	0,271475037	0,235979729	0,127335696	0,638107703	0,988361377	0,112756139	0,757103358	1,135555584	0,301922337
Fructose-6-P	(µg/mgFW)	0,040525322	0,047232703	0,061984563	0,053148189	0,052592867	0,078911257	0,063465819	0,082866794	0,063135992	0,069427049	0,094995371	0,084872699
Fumarate	(µg/mgFW)	0,615254167	1,570767393	0,76744209	0,793763485	1,380845975	0,400332292	0,039390695	0,974569827	0,083499729	0,054346968	1,057496475	0,344389608
GABA	(µg/mgFW)	0,009031061	0,007589702	0,007722618	0,010219087	0,00646922	0,008188049	0,011929247	0,0099624	0,012778406	0,012162392	0,011853842	0,008796118
Galactinol	(µg/mgFW)	6,60E-10	0,002282174	0,000977036	0,000612788	0,001126793	0,000668261	0,002860192	0,013184659	0,000252926	0,00959341	0,028656629	0,003001792
Galactonate	(µg/mgFW)	0,017308942	0,020723161	0,019830051	0,019456447	0,015955499	0,016127379	0,0124804	0,021412168	0,016592488	0,013102503	0,019882783	0,016743963
Galactose	(µg/mgFW)	0,003221173	0,004584256	0,002774372	0,003290311	0,00307201	0,002687021	0,005234686	0,008757526	0,002174393	0,007253789	0,006955656	0,004259326
Galactosylglycerol	(arbitrary/mgFW)	0,000274395	0,000535237	0,000285007	0,000388013	0,000314216	0,000350975	0,000725928	0,000971571	0,000547767	0,000941576	0,001228539	0,000646379
gamma-Tocopherol	(µg/mgFW)	0,001524386	0,000869922	0,001526003	0,001638219	0,000826591	0,001817093	0,001979963	0,001255267	0,002868438	0,002190116	0,001284213	0,001953483
Gentiobiose	(µg/mgFW)	0,001830768	0,000610916	0,000689934	0,001858109	0,000554109	0,001083704	0,002106923	0,00098621	0,000748739	0,002444033	0,000916624	0,00136044
Glutamine	(µg/mgFW)	0,194929589	0,192355128	0,237458019	0,218622554	0,167158161	0,222088789	0,147822509	0,105162514	0,320981118	0,095562617	0,076790675	0,228892641
Gluconate	(µg/mgFW)	0,002024707	0,002921002	0,001891153	0,002102478	0,001319393	0,001425282	0,001509197	0,003441129	0,00106002	0,001827241	0,004401466	0,00211299
Glucopyranose	(arbitrary/mgFW)	0,014907978	0,037803203	0,015718492	0,006740962	0,019805541	0,008680693	0,01825633	0,039786455	0,00905678	0,021037886	0,069783971	0,014567013
Glucopyranose(-H2O)	(arbitrary/mgFW)	0,001657433	0,001362822	0,000814236	0,00148995	0,001344444	0,001285343	0,001954895	0,001340238	0,00116873	0,001789959	0,00146753	0,001291727
Glucose	(µg/mgFW)	0,379926129	0,77340496	0,380344415	0,36015765	0,43364748	0,157507094	0,70915434	2,00319165	0,123121826	0,925402076	2,076497563	0,417506917
Glucose-6-P	(µg/mgFW)	0,096225468	0,104115208	0,130194922	0,125132758	0,111774169	0,167706311	0,145502407	0,176967634	0,13382186	0,156004118	0,225526395	0,177853474
Glucosyl-propylene glycol	(arbitrary/mgFW)	0,008189333	0,002515765	0,003482237	0,006912872	6,60E-10	0,002360554	0,00965128	0,002869569	0,003641171	0,009193948	0,002279366	0,002806821
Glutamate	(µg/mgFW)	0,333336694	0,365542419	0,344205751	0,339258699	0,315112858	0,285420589	0,238332262	0,305130487	0,476865494	0,21326666	0,249691987	0,245352748
Glycerate	(µg/mgFW)	0,013971362	0,048633587	0,036720444	0,016988045	0,036076448	0,024750529	0,008652112	0,038808805	0,014480779	0,00844897	0,066163343	0,02234516
Glycerate-3-P	(arbitrary/mgFW)	1,56069E-05	4,02243E-05	7,61236E-05	2,15061E-05	5,30697E-05	4,50131E-05	6,33169E-05	0,000108692	4,27839E-05	3,17861E-05	0,000108768	0,000165113
Glycerol	(µg/mgFW)	0,011909586	0,014176555	0,01437402	0,011919286	0,011254692	0,018308547	0,013849885	0,014924488	0,022745472	0,013766338	0,019022365	0,017906391
Glycerol-2-P	(arbitrary/mgFW)	0,000143066	0,0001544	0,000146842	0,000158602	6,22543E-05	0,000133776	0,00014119	0,000139552	0,000111501	0,000157519	0,000153987	0,000176707
Glycerol-3-P	(arbitrary/mgFW)	0,026852919	0,025982822	0,024776533	0,027049729	0,024916065	0,026964149	0,030044851	0,031392854	0,0261707	0,030508248	0,03054953	0,034237653
Glycine	(µg/mgFW)	0,004841125	0,012860896	0,013321536	0,003684516	0,007516685	0,01454182	0,003369602	0,007496556	0,026780217	0,001816355	0,006618121	0,011563926
Glycolate	(µg/mgFW)	0,001116343	0,002758349	0,002046634	0,001040861	0,001576953	0,00197845	0,001181424	0,001693408	0,002155808	0,000600241	0,001637343	0,001617704
Glycosylsalicylate	(arbitrary/mgFW)	0,000683927	0,000468553	0,001418916	0,000797122	0,000483968	0,001389037	0,000596375	0,00060549	0,001892951	0,000629074	0,000659978	0,000973322
Histidine	(µg/mgFW)	0,002015108	0,000865261	0,003956884	0,001269558	0,001283946	0,004211427	0,005776569	0,000675156	0,01480628	0,005161304	0,001708062	0,004107132
Homoserine	(arbitrary/mgFW)	3,26366E-05	4,75355E-05	4,09588E-05	4,11616E-05	4,66017E-05	3,54476E-05	1,81991E-05	1,9651E-05	4,12629E-05	1,58699E-05	1,99024E-05	2,74355E-05
Inositol-P-glycerol	(arbitrary/mgFW)	0,000165293	4,91626E-05	0,000304604	0,000105487	0,000274081	0,000187308	0,000288709	0,000388795	7,70418E-05	0,000371811	0,000351285	0,000731574
Isoleucine	(µg/mgFW)	0,002614684	0,002154663	0,001895256	0,00215552	0,0017224	0,003695453	0,008267353	0,002945938	0,0098211	0,006056888	0,003571832	0,004596248
Isothiocyanoethylphosphate	(arbitrary/mgFW)	0,000150901	0,000229191	0,000189677	7,27685E-05	0,000156767	0,000184591	8,75634E-05	0,000342209	0,000119049	9,04027E-05	0,000298912	0,000183882
Leucine	(µg/mgFW)	0,002585107	0,002413701	0,001687313	0,002389182	0,00164114	0,003189142	0,006202573	0,003386855	0,005301987	0,00494115	0,003913882	0,003235442
Linoleic acid	(arbitrary/mgFW)	0,001657241	0,002889611	0,001388986	0,002053801	0,001953483	0,001608638	0,001615336	0,001734298	0,001123134	0,001011479	0,001796806	0,001703709
Linolenic acid	(arbitrary/mgFW)	0,000798831	0,001195631	0,000747682	0,000857774	0,000946084	0,000939472	0,000647581	0,000765201	0,000787908	0,000514035	0,000873037	0,000944973
Lysine	(µg/mgFW)	0,003070047	0,002224656	0,003079951	0,002478638	0,001828015	0,005766097	0,009587788	0,002958313	0,011966166	0,008020157	0,003336507	0,005914623
Lyxonate	(arbitrary/mgFW)	0,000879279	0,000847779	0,00074554	0,000959137	0,000657827	0,000696231	0,00119216	0,001085007	0,000678939	0,001081861	0,001019872	0,000693541
Malate	(µg/mgFW)	0,539390102	0,865856294	0,637414677	0,608882637	0,789747864	0,500950397	0,043505657	0,239041302	0,12755558	0,042923887	0,270806821	0,206986984
Maleate	(arbitrary/mgFW)	0,003909303	0,005705607	0,003486214	0,004474568	0,004342915	0,003288101	0,001788527	0,003219298	0,001952367	0,001629423	0,002766302	0,003239406
Malonate	(µg/mgFW)	0,003664123	0,003843796	0,00424321	0,005016531	0,002725115	0,00319023	0,002027383	0,004569827	0,003310538	0,001964664	0,002814806	0,002616832
Maltose	(µg/mgFW)	0,002703851	0,003654186	0,001763845	0,003432109	0,006358006	0,001269074	0,012351197	0,0052189	0,001151689	0,006653359	0,009292419	0,001199462
Mannitol	(µg/mgFW)	0,006504984	0,000585879	0,001205747	0,007028342	0,000303603	0,00036074	0,007061839	0,000639944	0,001422945	6,60E-10	6,60E-10	0,000309866
Mannose	(µg/mgFW)	0,002293205	0,003144246	0,002455248	0,002391237	0,002071887	0,001966044	0,004071068	0,005183622	0,002139562	0,004668797	0,004361018	0,003155473
Melibiose	(µg/mgFW)	6,60E-10	0,000878014	0,000991859	6,60E-10	0,00072637	0,001018731	0,001307996	0,002227916	0,000922813	0,002237919	0,003643093	0,001484048
Methionine	(µg/mgFW)	0,001373096	0,001644386	0,001350068	0,001397475	0,001663579	0,001977942	0,001416628	0,000952283	0,003917351	0,000904749	0,001260318	0,001527905
Mimosine	(arbitrary/mgFW)	0,000114487	9,5635E-05	7,30744E-05	7,58551E-05	0,000123823	0,000126754	4,93454E-05	8,16266E-05	7,09088E-05	5,44083E-05	7,10019E-05	5,24082E-05
Monomethylphosphate	(arbitrary/mgFW)	2,32502E-05	4,09207E-05	5,25193E-05	4,16263E-05	2,37068E-05	2,41397E-05	2,26667E-05	8,4986E-05	1,96002E-05	3,40718E-05	4,28692E-05	3,62453E-05
Monopalmitine	(arbitrary/mgFW)	0,001413668	0,002479295	0,001768416	0,001698708	0,001333422	0,001769063	0,001319624	0,002343258	0,00124663	0,001287501	0,002596707	0,001938157
Myo-Inositol	(µg/mgFW)	0,023042933	0,062577478	0,030461908	0,039801498	0,053553756	0,032392886	0,050952415	0,145037956	0,01538102	0,09254895	0,188294232	0,071307835
myo-Inositol-1-P	(arbitrary/mgFW)	0,003926792	0,002993286	0,004202948	0,003564583	0,003820633	0,007402655	0,004517917	0,00509674	0,006705347	0,005525811	0,006604854	0,009531186
Nicotinate	(arbitrary/mgFW)	0,000713532	0,000583698	0,000712494	0,000712881	0,000539184	0,000825146	0,000578975	0,000502544	0,000713998	0,000641554	0,000664991	0,000855427
O-Acetyl-Serine	(µg/mgFW)	0,001598121	0,001421746	0,001759638	0,001582834	0,001452428	0,001607601	0,001958262	0,001964713	0,001620429	0,001794074	0,002109855	0,002205509
Pantothenate	(arbitrary/mgFW)	0,000193037	0,000148156	0,000233431	0,000242237	0,000136798	0,000192452	0,000121387	0,000169705	0,000242766	0,000177405	0,000228266	0,000317825
Pentonate-4	(arbitrary/mgFW)	0,000306096	0,000335313	0,000335101	0,000324306	0,000199058	0,000496315	0,000186384	0,000421458	0,000699141	0,000243528	0,000457395	0,000366592
Phenylalanine	(µg/mgFW)	0,00396727	0,00388264	0,004942344	0,00398159	0,003154092	0,007103854	0,009356875	0,004266191	0,022201137	0,006913551	0,005069195	0,007327002
Phosphate	(µg/mgFW)	0,36837483	0,299544426	0,479499519	0,387821604	0,321921692	0,736891836	0,52109096	0,434059752	0,905303268	0,506758668	0,600583872	0,742485936
Phyllodihydroquinone	(arbitrary/mgFW)	2,07313E-05	2,48704E-05	8,19653E-06	1,49656E-05	1,60825E-05	2,06436E-05	2,32496E-05	1,87892E-05	1,53399E-05	4,097E-05	2,86616E-05	2,78913E-05
Phytol-2	(arbitrary/mgFW)	0,008613469	0,013167975	0,006898138	0,009153465	0,010221347	0,008881065	0,009797266	0,011340488	0,008825976	0,007614528	0,008574139	0,00814676
Pipecolate	(µg/mgFW)	0,001461519	0,000464032	0,002114535	0,001402942	0,00046594	0,000847977	0,001296449	0,000367179	0,002966478	0,000984465	0,000583278	0,000485806
Proline	(µg/mgFW)	0,023033484	0,02596175	0,016685336	0,022181323	0,025718037	0,018844526	0,026658559	0,035826063	0,030250532	0,030494812	0,035071934	0,028623743
Putrescine	(µg/mgFW)	0,009547395	0,015078814	0,041544534	0,007211309	0,01741092	0,057744615	0,029660817	0,024094315	0,094823319	0,027712001	0,017719157	0,055564477
Pyruvate	(µg/mgFW)	0,006000432	0,023444606	0,007732457	0,023675433	0,022636423	0,003299521	6,60E-10	0,005887485	0,001355022	6,60E-10	0,00822427	0,001631511
Raffinose	(µg/mgFW)	0,004013936	0,010655322	0,005192023	0,005551228	0,006136305	0,003863451	0,02159731	0,048946054	0,002012843	0,055873863	0,103267103	0,014467962
Rhamnose	(µg/mgFW)	0,000718203	0,00081088	0,000718535	0,000713774	0,000705399	0,000864652	0,000836686	0,000902849	0,000997107	0,000829185	0,000966059	0,000918061
Ribonate	(arbitrary/mgFW)	0,001598056	0,001620576	0,001509283	0,001745845	0,001252314	0,001374998	0,001118767	0,001669082	0,001144991	0,001059081	0,001580414	0,001293241
Ribose	(µg/mgFW)	0,000909928	0,00086795	0,000757771	0,000858672	0,000685577	0,000917799	0,001328303	0,001123369	0,000917309	0,001405064	0,001382915	0,001085089
Ribose-5-P	(µg/mgFW)	6,60E-10	0,000118406	6,60E-10	6,60E-10	6,60E-10	6,60E-10	0,00027264	6,60E-10	0,000137308	6,60E-10	0,000265927	6,60E-10
Ribulose-5-P	(µg/mgFW)	6,60E-10	0,002752208	0,003211167	6,60E-10	0,003225728	0,003870685	0,005700652	0,005368078	0,003251554	0,006955316	0,008240959	0,005024843
Salicylate	(µg/mgFW)	0,000121599	6,40249E-05	0,000137396	0,000110521	8,3772E-05	0,000131947	9,88636E-05	5,32908E-05	0,000199337	9,85736E-05	0,00017749	0,000124476
Serine	(µg/mgFW)	0,017862366	0,009900481	0,031134428	0,018031148	0,006914458	0,038439467	0,091509831	0,043687144	0,113911332	0,197332097	0,038745092	0,05513105
Shikimate	(µg/mgFW)	0,005470261	0,007686614	0,005785459	0,006357502	0,006931432	0,004684898	0,002442523	0,005343888	0,002528692	0,0026886	0,005834695	0,004312004
Sinapinate-cis	(arbitrary/mgFW)	0,00118121	0,001084152	0,000916553	0,000948421	0,000510576	0,001093585	0,000507886	0,001527649	0,000835072	0,000995332	0,001656523	0,001243914
Sinapinate-trans	(arbitrary/mgFW)	0,051766624	0,066577741	0,049676407	0,055620838	0,05520196	0,05166726	0,027594761	0,05403535	0,037008878	0,031346628	0,052821235	0,056695239

Sorbitol	(µg/mgFW)	0,012464101	0,001213553	0,002389876	0,013430669	0,000420675	0,000349935	0,013420769	0,001319626	0,00279098	0,014024362	6,60E-10	0,000351886
Spermidine	(µg/mgFW)	0,00214169	0,001502525	0,002317756	0,001359865	0,000639725	0,003348912	0,002041631	0,00090083	0,004177275	0,001755165	0,001029527	0,002623143
SSA	(µg/mgFW)	0,001790186	0,001584301	0,001692389	0,002237167	0,001121603	0,000640027	0,001772146	0,001657505	0,001363531	0,001748301	0,001163432	0,001015908
Stigmasterol	(µg/mgFW)	0,003025896	0,002341964	0,002733943	0,003088154	0,00162049	0,006213199	0,004863536	0,002349375	0,005322763	0,007803373	0,004880349	0,005714459
Succinate	(µg/mgFW)	0,013444607	0,030784253	0,018196425	0,0179784	0,030791031	0,011052418	0,005238234	0,013231948	0,010244802	0,005153055	0,014471561	0,006895838
Sucrose	(µg/mgFW)	0,828163604	1,023756433	0,93928035	1,071373264	0,657270701	0,842097143	1,667304704	1,863431834	0,773978009	1,720124507	2,525581066	1,22361298
Threitol	(arbitrary/mgFW)	0,000352371	0,000152415	0,000283768	0,00027699	6,94466E-05	0,000136852	0,000302679	0,000206052	0,000332036	0,000230985	0,000168998	0,000132948
Threonate	(arbitrary/mgFW)	0,001632321	0,001954396	0,001828839	0,001988023	0,001536212	0,001178117	0,001029643	0,001468214	0,001041027	0,001107348	0,001524361	0,001359823
Threonate-lactone	(arbitrary/mgFW)	0,000678291	0,000717279	0,000624153	0,000741486	0,000526804	0,00042174	0,000493899	0,000577031	0,000352148	0,000539146	0,000659534	0,000515075
Threonine	(µg/mgFW)	0,01692962	0,007445723	0,009440168	0,009356531	0,004477415	0,014979215	0,01993653	0,010515777	0,028248167	0,033382187	0,014185117	0,017850947
Trehalose	(µg/mgFW)	0,001089936	0,001513113	0,001188139	0,002224939	0,000607135	0,000969096	0,000717961	0,001029301	6,60E-10	0,002120503	0,001637789	0,00233688
Tryptophan	(µg/mgFW)	0,001632979	0,001285298	0,001816949	0,001525154	0,000885759	0,002493829	0,004037797	0,001434257	0,00822366	0,003480902	0,00238776	0,002711694
Tyramine	(arbitrary/mgFW)	0,001366034	0,001446083	0,001238663	0,001323189	0,00119098	0,00124518	0,002373918	0,002036638	0,001839563	0,002310706	0,001931322	0,00169035
Tyrosine	(µg/mgFW)	0,001354589	0,001235545	0,001273634	0,001340295	0,000930855	0,002071739	0,00335479	0,002569305	0,004277259	0,003520273	0,00325135	0,002576462
Urate	(arbitrary/mgFW)	0,001017058	0,001367298	0,003043468	0,001252362	0,000923977	0,00297644	0,0014192	0,001780836	0,003311505	0,001705085	0,001684741	0,003963076
Valine	(µg/mgFW)	0,003817474	0,003965822	0,003596134	0,004122208	0,003203608	0,005211358	0,007022605	0,004562396	0,011378049	0,007097445	0,005772867	0,005806582
Xanthine	(arbitrary/mgFW)	9,67024E-05	7,29303E-05	0,000108358	0,000173691	6,03773E-05	9,54025E-05	0,00013771	0,000105473	0,000120269	0,000122254	8,00048E-05	0,000113461
Xylose	(µg/mgFW)	0,00292529	0,00275626	0,001998952	0,003144433	0,001842603	0,001954026	0,004723949	0,005125647	0,001598329	0,005882202	0,006129462	0,002772356
